# CD14 Signaling Restrains Chronic Inflammation through Induction of p38-MAPK/SOCS-Dependent Tolerance

**DOI:** 10.1371/journal.ppat.1000687

**Published:** 2009-12-11

**Authors:** Bikash Sahay, Rebeca L. Patsey, Christian H. Eggers, Juan C. Salazar, Justin D. Radolf, Timothy J. Sellati

**Affiliations:** 1 Center for Immunology and Microbial Disease, Albany Medical College, Albany, New York, United States of America; 2 Department of Medicine, University of Connecticut Health Center, Farmington, Connecticut, United States of America; 3 Connecticut Children's Medical Center, Division of Pediatric Infectious Diseases, Hartford, Connecticut, United States of America; 4 Department of Genetics and Developmental Biology, University of Connecticut Health Center, Farmington, Connecticut, United States of America; Medical College of Wisconsin, United States of America

## Abstract

Current thinking emphasizes the primacy of CD14 in facilitating recognition of microbes by certain TLRs to initiate pro-inflammatory signaling events and the importance of p38-MAPK in augmenting such responses. Herein, this paradigm is challenged by demonstrating that recognition of live *Borrelia burgdorferi* not only triggers an inflammatory response in the absence of CD14, but one that is, in part, a consequence of altered PI3K/AKT/p38-MAPK signaling and impaired negative regulation of TLR2. CD14 deficiency results in increased localization of PI3K to lipid rafts, hyperphosphorylation of AKT, and reduced activation of p38. Such aberrant signaling leads to decreased negative regulation by SOCS1, SOCS3, and CIS, thereby compromising the induction of tolerance in macrophages and engendering more severe and persistent inflammatory responses to *B. burgdorferi*. Importantly, these altered signaling events and the higher cytokine production observed can be mimicked through shRNA and pharmacological inhibition of p38 activity in CD14-expressing macrophages. Perturbation of this CD14/p38-MAPK-dependent immune regulation may underlie development of infectious chronic inflammatory syndromes.

## Author Summary

Macrophages express CD14 which partners with Toll-like receptor 2/1 to recognize bacterial lipoproteins such as those of *Borrelia burgdorferi*, the causative agent of Lyme disease. *In vitro* evidence demonstrates that blocking CD14 recognition of bacterial components ablates innate host cell inflammatory responses. Similarly, blocking downstream p38 kinase activity dampens the cellular response to these same microbial stimuli. This body of work underpins two well-established paradigms which cite the primacy of CD14 in facilitating TLR recognition of microbes to initiate proinflammatory signaling events and the importance of p38 in augmenting such responses. However, contrary to these paradigms, our prior study using a mouse model of Lyme disease demonstrated an association between CD14 deficiency, increased bacterial burden, and more severe and persistent disease. Herein, we provide a mechanistic explanation for this unanticipated host immune response implicating impaired negative regulation of inflammatory signaling pathways as an underlying cause. Consequent to impaired negative regulation the host becomes “intolerant” of continued exposure to bacteria and thus mounts a perpetual inflammatory response to their presence. An intriguing question raised by these findings is whether individual differences in the severity and clinical course of infection might reflect the susceptibility of the patient's innate immune system to tolerization.

## Introduction

Toll-like receptor (TLR) signaling orchestrates the innate response to danger-associated molecular patterns (DAMPs) associated with pathogens and/or select endogenous molecules. The principal proinflammatory DAMPs of the spirochetal pathogen *Borrelia burgdorferi*, the causative agent of Lyme disease, are triacylated lipoproteins recognized by heterodimers of TLR2 and TLR1 [1]. Such recognition by TLR2 activates the NF-κB, PI3K/AKT, and MAPK pathways which coordinately regulate inflammation-associated gene activities responsible for host defense [2]. Although many aspects of Lyme disease pathogenesis remain ill-defined, it generally is accepted that clinical manifestations result primarily, perhaps entirely, from the host's immune response to spirochetes [3]. In this latter regard, borrelial lipoproteins are thought to be the chief elicitor of inflammation.

CD14, a GPI-anchored protein expressed by macrophages (MΦ) and neutrophils, facilitates pro- and anti-inflammatory cytokine production in response to a variety of DAMPs. Mice deficient for CD14 and their MΦ exhibit hyporesponsiveness when exposed to DAMPs in the form of a bacterial lysate or purified agonists such as LPS, lipoproteins, and their synthetic analogs [4]–[6]. This hyporesponsiveness has been attributed to the lower affinity of non-CD14-complexed LPS for TLR4 [7], the requirement for CD14 in MyD88-independent signaling [8], and/or the inability of p38, a member of the serine/threonine MAPK family, to be induced in the absence of CD14 [9]. While the majority of the literature suggests that CD14 is indispensable for elaboration of an inflammatory response to its cognate DAMPs [4]–[6], we previously reported that both *in vitro* and *in vivo* recognition of *B. burgdorferi* in the absence of CD14 leads to exaggerated proinflammatory cytokine production and worsening disease [10]. However, in contrast to live bacteria, an equivalent number of lysed spirochetes are approximately 100-fold less stimulatory and are primarily dependent upon CD14 to initiate an inflammatory response from MΦ [10].

At the point of contact between a pathogen and the MΦ plasma membrane molecules such as CD14, TLRs, and phagocytic receptors coalesce into cholesterol-rich detergent-insoluble/detergent-resistant domains or “lipid rafts” [11]. These platforms serve as an initiating site for innate immune signaling cascades and bacterial entry into the phagocyte [12]–[15]. CD14, like other GPI anchored proteins, may participate in cellular signaling either by direct association with transmembrane spanning proteins [16] and/or through regulation of the charge character of the inner leaflet of the plasma membrane which alters recruitment of cellular proteins to the lipid-rich domain [17]–[19]. Following exposure of host cells to pathogens or their isolated constituents, p38 is activated through phosphorylation [20]. After microbial uptake, the action of p38 drives maturation of the phagosome [21], activates downstream kinases that result in the nuclear translocation of NF-κB [22], and stabilizes cytokine mRNA [23]. Because the pleiotropic action of p38 is thought to augment inflammation, the pharmaceutical industry has actively pursued development of p38 inhibitors for the treatment of inflammatory disorders [20]. However, through the induction of tolerance [24], p38 also has an anti-inflammatory role to play by virtue of its ability to induce suppressors of cytokine signaling 3 (SOCS3) and IL-10 [25] which negatively regulates pathogen-induced inflammation [26]. Tolerance represents a state of MΦ unresponsiveness to perpetual exposure to bacterial stimuli [27],[28].

Herein, we advance a mechanistic explanation for how CD14 regulates the intensity and duration of host responses to bacterial challenge which distinguishes CD14-dependent from -independent signaling and recognition of live versus lysed *B. burgdorferi*. Using a mouse model of Lyme disease, we show that *B. burgdorferi*-activated CD14^−/−^ MΦ release significantly more proinflammatory cytokines and do so in a manner only partially dependent on TLR2. Initial engagement of receptors (*i.e.*, TLR2 and/or non-TLR2) on the surface of CD14^−/−^ MΦ by *B. burgdorferi* leads to the accumulation of PI3K in lipid rafts resulting in higher phospho-AKT levels, lower p38 activation, decreased SOCS activity, and increased and persistent inflammation. The perpetual nature of CD14^−/−^ MΦ responses to spirochetes is a reflection of the cell's inability to be tolerized via CD14/p38-dependent SOCS induction. Importantly, inhibition of PI3K in CD14^−/−^ MΦ restores p38 activity to wild-type levels and thus reduces TNF-α release in response to *B. burgdorferi*. Additionally, inhibition of p38 has the commonly observed effect of suppressing proinflammatory responses, but only when cells are exposed to lysed bacteria and not when whole organisms are used as a stimulus. Collectively, our findings challenge the current paradigm by supporting the rather provocative notion that CD14 signaling: i) is entirely dispensable for elaboration of a proinflammatory response to DAMPs other than lipoproteins, ii) is essential for prolonged p38 activation, and iii) serves a critical function in resolution of inflammation through induction of tolerance.

## Results

### CD14 deficiency augments TLR-dependent gene activity

**Figure 1:**
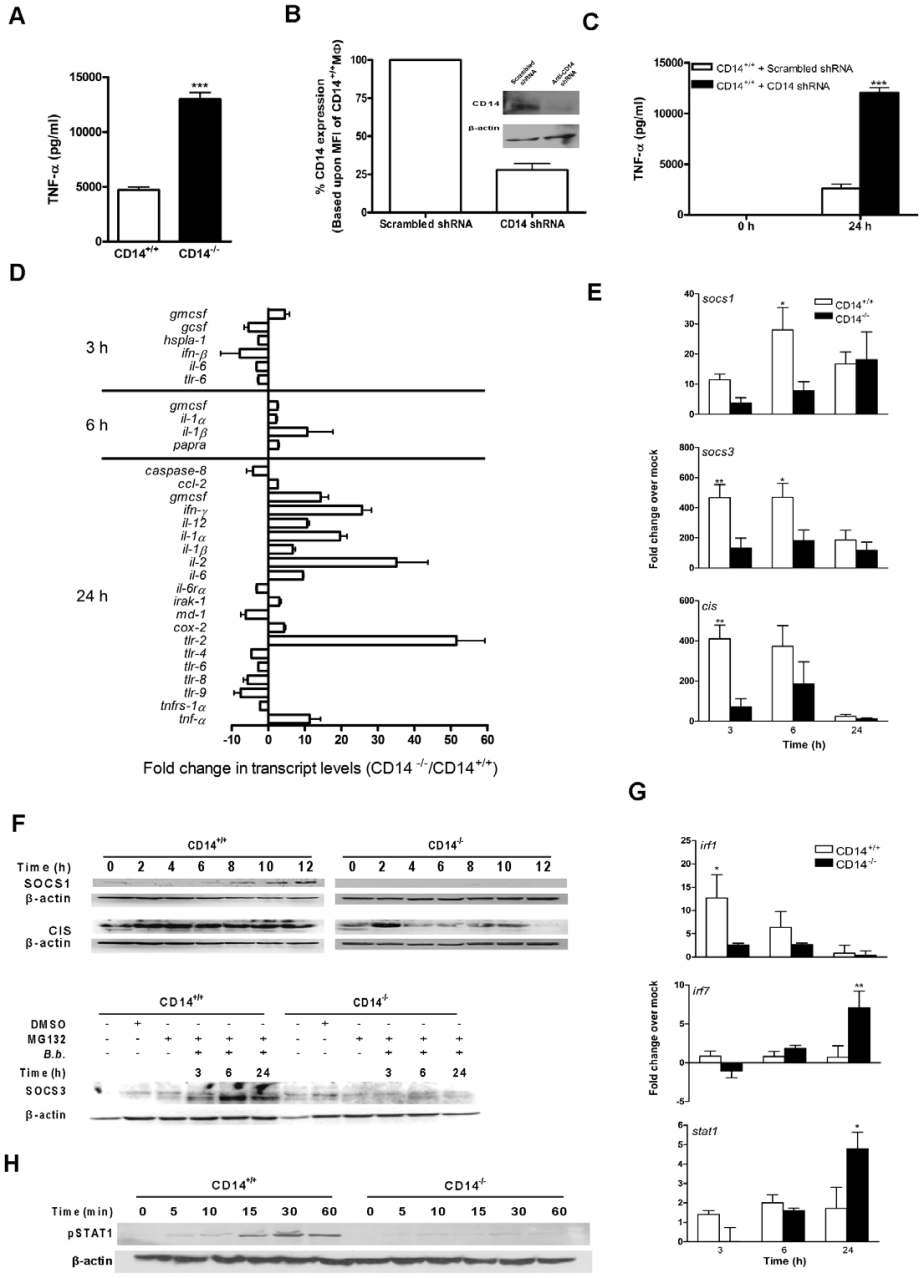
Higher *B. burgdorferi*-induced inflammatory gene activity is associated with lower SOCS levels in CD14^−/−^ MΦ. A) MΦ isolated from CD14^+/+^ and CD14^−/−^ mice were incubated with *B. burgdorferi* at a MOI of 10 for 24 h and TNF-α levels in culture supernatant were measured by CBA. B) Lentiviral transduction was used to knock down CD14 in MΦ as determined by a reduction in mean fluorescent intensity (MFI) and by Western blot analysis (inset). C) The lentivirus-treated MΦ were incubated with *B. burgdorferi* for 24 h and TNF-α levels were measured as for (A). D) MΦ isolated from CD14^+/+^ and CD14^−/−^ mice were incubated with *B. burgdorferi* for 3, 6 and 24 h. Total RNA was isolated and used to perform qPCR for simultaneous interrogation of 84 genes associated with TLR signaling. The results presented are the ratio of fold change in HPRT-normalized gene activity in CD14^−/−^ versus CD14^+/+^ MΦ and error bars represent SEM calculated on the basis of three independent experiments. E) Total RNA isolated from CD14^+/+^ and CD14^−/−^ MΦ incubated with *B. burgdorferi* was analyzed by qPCR for *socs1*, *socs3* and *cis*. Results are presented as fold change over respective mock-infected control and were normalized with 18S rRNA transcript. F) Equivalent protein from lysed MΦ were separated by 12% SDS-PAGE, transferred to a PVDF membrane and probed with antibodies directed against SOCS1, SOCS3, CIS or β-actin. G) Total RNA isolated from CD14^+/+^ and CD14^−/−^ MΦ incubated with *B. burgdorferi* was analyzed by qPCR for *irf1*, *irf7* and *stat1*. Dotted lines denote the 2-fold over mock change considered significant for the purposes of interpretation. H) Equivalent protein from lysed MΦ were subjected to Western blot analysis using antibodies directed against phospho-specific STAT1 or STAT3, or β-actin. Results represent mean±SEM from three to four independent experiments. *P<0.05, **P<0.01, ***P<0.001.

Compared to CD14^+/+^ MΦ, activation of CD14^−/−^ cells by *B. burgdorferi* results in greater transcription and persistent surface expression of TLR2 and a concomitant increase in proinflammatory cytokine production, particularly TNF-α ([10] and Figure 1A). To rule out the potential influence of developmental defects introduced through genetic mutation of *cd14*, expression of CD14 by MΦ was transiently targeted through use of shRNA. A pRS-shGFP lentiviral expression vector was used to transduce either a CD14 shRNA or a scrambled control shRNA into CD14^+/+^ MΦ. At 3 days p.i., a 70% reduction in the MFI of CD14 expression was observed by flow cytometry (Figure 1B), an effect confirmed by Western blot analysis (Inset). After incubation with *B. burgdorferi*, CD14 shRNA-expressing cells produced markedly higher TNF-α (Figure 1C), a trend identical to that observed with *B. burgdorferi*-activated CD14^−/−^ MΦ (Figure 1A).

To more broadly evaluate the impact of CD14 deficiency on the MΦ inflammatory transcriptome, we measured gene activity associated with TLR signaling. Greater transcription of interleukins (*i.e.*, *il1α*, *il1β*, *il2*, *il6*, and *il12*), interferons (*i.e.*, *ifnγ*), chemokines (*i.e.*, *ccl2*), growth factors (*i.e.*, *g-csf*), and proinflammatory lipid mediators (*i.e.*, *cox2*) was observed in CD14^−/−^ than in CD14^+/+^ MΦ (Figure 1D); many of these genes are associated with the NF-κB signaling pathway. TLR2 transcript levels, but not those of other TLRs, were elevated 50-fold in CD14^−/−^ MΦ compared to wild-type cells. Gene induction in CD14^+/+^ MΦ peaked at 3 h and returned to baseline by 24 h p.i.. In contrast, CD14^−/−^ cells more actively transcribed these genes at 3 h and did so throughout the course of the experiment (data not shown), suggesting a critical role for CD14 in downmodulation of inflammation.

### *B. burgdorferi* fails to trigger expression of negative regulators of TLR signaling in the absence of CD14

The above findings prompted an evaluation of the impact of CD14 deficiency on negative regulators of TLR signaling which include competitive inhibitors (*e.g.*, IRAKM), protein phosphatases [*e.g.*, MAPK phosphatase 1 (MKP1)], and E3 ligases (*e.g.*, SOCSs) [29]. *B. burgdorferi* induced significantly higher transcription of *socs1* [∼3.5-fold by 6 h p.i. (*P*<0.05)], s*ocs3* [∼3.5-fold by 3 h and ∼2.5-fold by 6 h (*P*<0.01)], and *cis* [∼5.6-fold by 3 h (*P*>0.01)] in CD14^+/+^ cells compared to cells lacking CD14 (Figure 1E). However, by 24 h p.i. transcription of these negative regulators in CD14^+/+^ and CD14^−/−^ MΦ was indistinguishable. Transcription of *mkp1* and *irakm* was not significantly different in these cells at any time point studied (data not shown). Consistent with their respective transcript levels, increased expression of SOCS1 and CIS was seen in CD14^+/+^ MΦ, whereas little or no expression was detected in their CD14-deficient counterparts (Figure 1F). Upon inhibition of proteosome-mediated degradation of SOCS3 using MG132 (10 µM), SOCS3 expression only was observed in CD14^+/+^ cells (Figure 1F). Collectively, these findings suggest a critical role for CD14 in negative regulation of TLR signaling and implicate SOCS family members in this process.

To discover why negative regulation of TLRs is diminished in CD14^−/−^ cells, we examined the expression of three transcription factors known to directly or indirectly regulate transcription of *socs1*, *socs3*, and *cis* [30],[31]. *B. burgdorferi* induced significantly higher transcription of *irf1* in CD14^+/+^ than in CD14^−/−^ MΦ (*P*<0.05) whereas transcription of both *irf7* and *stat1* was significantly higher in CD14^−/−^ MΦ (*P*<0.01 and 0.05, respectively) (Figure 1G). No differences were seen for *irf3*, *stat3*, or *stat4* (data not shown). Additionally, Western blot analysis revealed that levels of phospho-STAT1 (Figure 1H), but not phospho-STAT3 (data not shown), were higher in CD14^+/+^ than in CD14^−/−^ MΦ. Given their capacity to upregulate SOCS, the lower *irf1* transcript levels and STAT1 phosphorylation in CD14^−/−^ MΦ is consistent with reduced SOCS expression observed in these cells.

### The inflammatory response to *B. burgdorferi* is perpetuated in the absence of CD14

**Figure 2:**
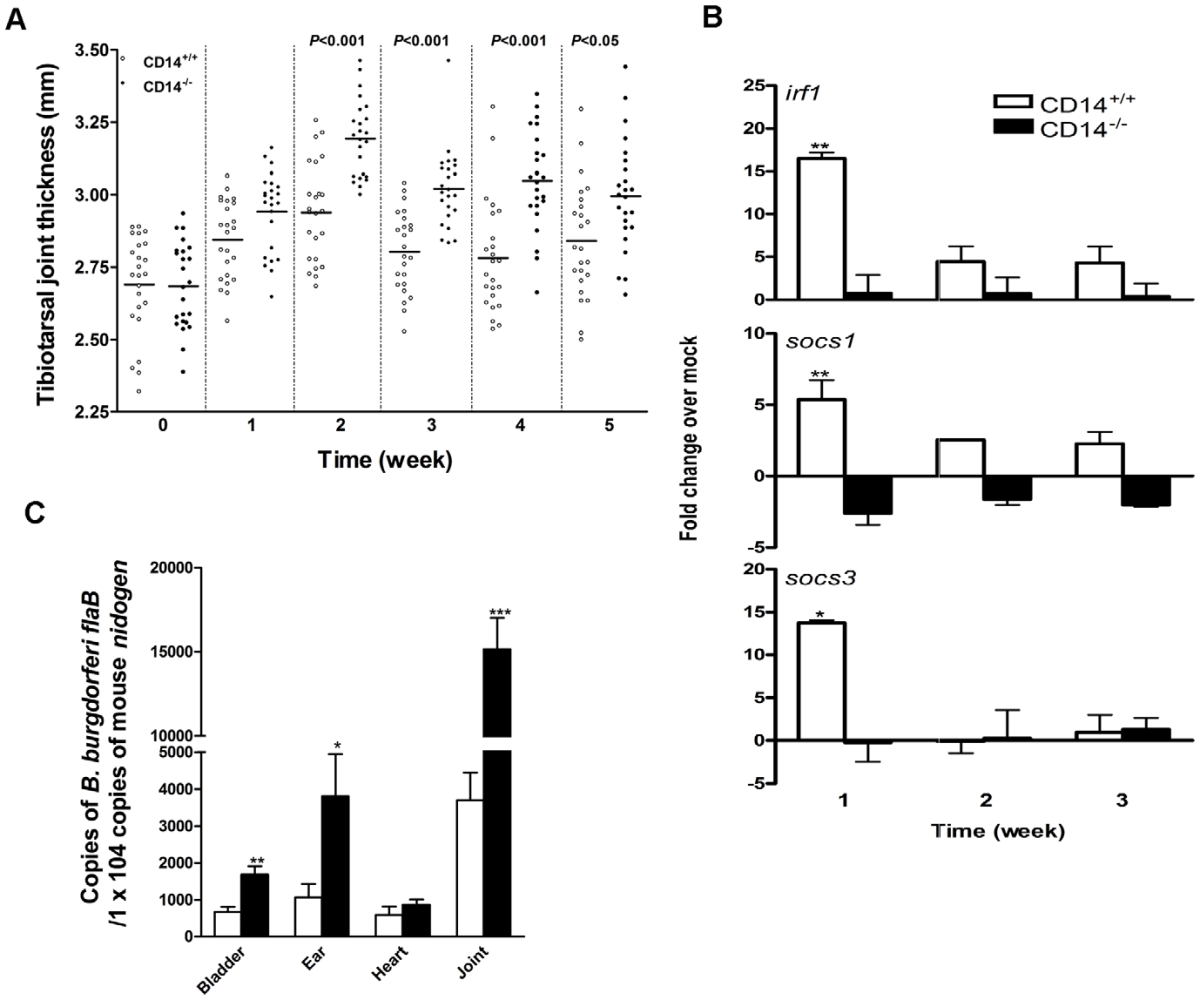
CD14 deficiency is associated with greater inflammation and higher bacterial burden with reduced *socs* transcription in response to *B. burgdorferi*. A) CD14^+/+^ and CD14^−/−^ mice were infected with 1×10^5^ *B. burgdorferi* and tibiotarsal joint thickness was measured at 1 week intervals. The horizontal bars indicate mean thickness for each group and the data are representative of two independent experiments (n = 24). B) Total RNA was isolated from the tibiotarsal joints of CD14^+/+^ and CD14^−/−^ mice (n = 6), an equal amount of RNA was pooled from each joint and 0.5 µg of pooled RNA was used for preparing cDNA. The cDNA was analyzed by qPCR to determine the levels of *irf1*, *socs1* and *socs3* transcript. Results represent mean±SEM from two independent experiments wherein samples were run in triplicate. *P<0.05, **P<0.01. C) DNA from the indicated organs was collected 6 weeks p.i. and the bacterial burden was determined using Taqman® probes for *flaB* of *B. burgdorferi*; murine *nidogen* served as a control. Results represent mean±SEM from two independent experiments (n = 24) wherein samples were run in triplicate. *P<0.05, **P<0.01.

The mouse model of Lyme disease was used to elucidate the relationship between CD14 signaling, SOCS activity, and disease progression. Arthritis, an inflammatory hallmark of Lyme disease, is a self-limiting process in immunocompetent mice that peaks between 2 and 3 weeks p.i. and typically resolves by 6 weeks [32]. Syringe inoculation of mice with *B. burgdorferi* resulted in tibiotarsal joint swelling in CD14^+/+^ and CD14^−/−^ mice which peaked at 2 weeks p.i.. Distinct from CD14^+/+^ joints wherein swelling (which grossly correlates with arthritis progression) resolved by 3 to 4 weeks p.i., CD14^−/−^ joints were more inflamed than their wild-type counterparts at each time point and remained swollen for the duration of the experiment (Figure 2A). Consistent with the more severe and prolonged joint swelling in CD14^−/−^ mice, transcription of *irf1*, *socs1*, and *socs3* in joint tissue was greatly reduced compared to wild-type mice (Figure 2B). Given the elevated cytokine production (*e.g.*, TNF-α and IFN-γ, Figure S3 and [10]) at one week in *B. burgdorferi*-infected CD14^−/−^ mice, one might have predicted decreased spirochete numbers through enhanced antimicrobial MΦ effector functions later (at 6 weeks) in the disease process; surprisingly, all organs recovered from CD14^−/−^ mice at this time point, with the exception of the heart, had heavier bacterial burdens than those of wild-type animals (Figure 2C). A similar increase in Lyme disease susceptibility characterized by increased tibiotarsal joint thickness (Figure S1), enhanced production of pro-inflammatory cytokines (Figure S2), and greater bacterial burden (Table S1) also was observed in CD14^−/−^ C57BL/6 mice. These results are particularly noteworthy as C57BL/6 mice are highly resistant to the development of Lyme pathology [33], and corroborate the importance of CD14 in host immunity irrespective of inherent differences in disease susceptibility.

### Killing, but not internalization, of *B. burgdorferi* by MΦ is impaired in the absence of CD14

**Figure 3:**
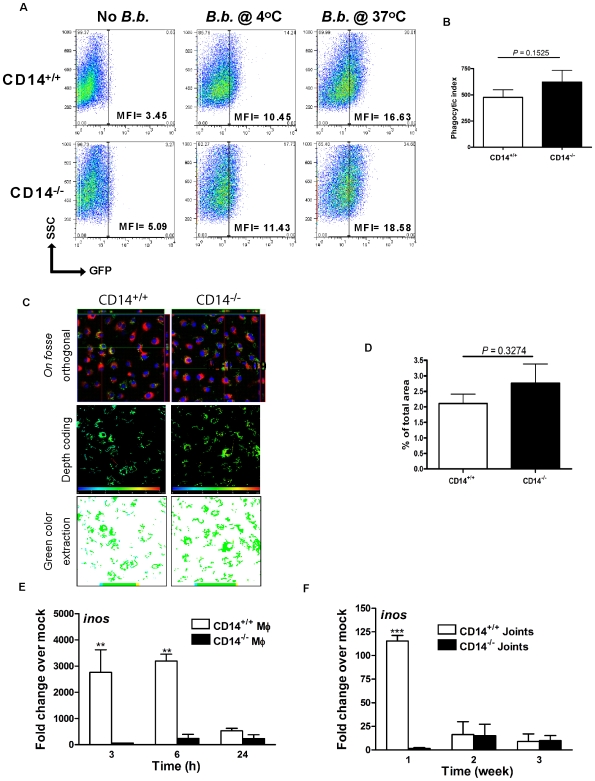
*B. burgdorferi*-induced transcription of *inos*, but not phagocytic uptake of spirochetes, is impaired by CD14 deficiency. A) MΦ were incubated with GFP-expressing *B. burgdorferi* at a MOI of 10 at 4°C and 37°C for 6 h. Phagocytosis of *B. burgdorferi* was determined by flow cytometry. B) Phagocytic indices were calculated based upon the results of five independent flow cytometric experiments. C) MΦ were incubated with GFP-expressing *B. burgdorferi* at a MOI of 100 for 6 h and the cytoplasm and nucleus were stained with wheat-germ agglutinin-Alexa Fluor 647 and DAPI, respectively. Optical sections were collected at every 0.5µm and were used to generate orthogonal sections (upper panel), depth coded images (middle panel) and green color extraction (lower panel) (see Methods). D) The green area of 10 randomly chosen fields from both genotypes was used for statistical analysis. E) *inos* transcript levels were determined by qPCR using RNA isolated from MΦ incubated with *B. burgdorferi* or F) using RNA pooled from joints isolated from infected mice, as described in Figure 2. Results represent mean±SEM from two to five independent experiments. **P<0.01, ***P<0.001.

To determine whether the hyper-responsiveness of CD14^−/−^ MΦ (Figure 1A) and/or increased bacterial burden in CD14^−/−^ mice (Figure 2C) reflected differences in binding and uptake of spirochetes we evaluated phagocytosis using GFP-expressing *B. burgdorferi*. The role of CD14 in the binding and/or internalization of spirochetes by MΦ was evaluated by flow cytometry and two-photon laser scanning confocal microscopy. CD14^+/+^ and CD14^−/−^ MΦ were incubated at 4°C and 37°C for 6 h with or without GFP-labeled *B. burgdorferi* at a MOI of 10; this allowed for discrimination between binding of the spirochete to the cell surface and active phagocytosis. At 4°C, a temperature which impairs bacterial uptake, the absence of CD14 had no effect on either the percentage of cells positive for GFP or the MFI of the positive population (Figure 3A). Similarly, at 37°C, a temperature permissive for uptake of bacteria, MΦ of both genotypes bound and phagocytosed an equivalent number of spirochetes. Comparable results were obtained when a MOI of 100 was used (data not shown). The phagocytic index was calculated as described elsewhere [34], and confirmed that the absence of CD14 does not alter the capacity of MΦ to bind and internalize *B. burgdorferi* (Figure 3B). Flow cytometry results were corroborated through visualization of spirochetes bound to the cell surface and found within the cell's interior using two-photon laser confocal microscopy. For these experiments a MOI of 100 was used to increase the number of organisms per field. The distribution of spirochetes associated with CD14^+/+^ and CD14^−/−^ MΦ was identical as determined by color depth coding analysis of images of spirochetes (which are green) within the middle optical sections of the Z-stack of cells (Figure 3C) and as seen in entire optical Z-stack for wild-type and CD14^−/−^ MΦ (Video S1 and S2, respectively). The amount of green signal as a percentage of the total area represents a measure of internalized spirochetes and was found to be similar in both CD14^+/+^ and CD14^−/−^ MΦ (Figure 3D). Though more sparsely populated with spirochetes, similar results were obtained visualizing MΦ monolayers incubated with *B. burgdorferi* using a MOI of 10 (data not shown).

Although differences in binding and phagocytosis were not observed, the antimicrobial effector functions of CD14^+/+^ and CD14^−/−^ MΦ might differ and thus explain the differential *in vivo* bacterial burden (Figure 2C). Killing of bacteria within the phagolysosomal compartment depends, at least in part, on the production of RNS/ROS [35],[36]. Assessing gene activity both *in vitro* and *in vivo* it was observed that *inos* transcript levels were significantly higher in cells and joints from CD14^+/+^ mice (Figure 3E and F, respectively). Though not directly linked experimentally these differences might underlie the two-fold more culturable spirochetes being recovered from 6 h-infected CD14^−/−^ (7,252 bacteria/ml) versus CD14^+/+^ (3,567 bacteria/ml) MΦ as measured using a modified tissue culture-infective dose method. In consequence, prolonged survival of *B. burgdorferi* internalized by CD14^−/−^ MΦ might correlate with their persistence *in vivo*.

### CD14 signaling regulates activation of the p38-MAPK pathway

**Figure 4:**
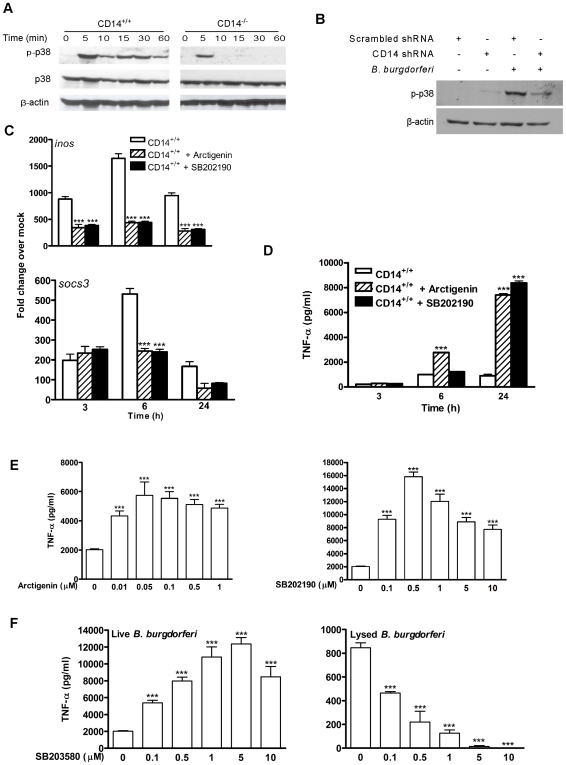
CD14 deficiency results in dysregulated p38-MAPK signaling and cytokine production in response to *B. burgdorferi*. A) Equal protein from lysates of CD14^+/+^ and CD14^−/−^ MΦ incubated with *B. burgdorferi* were separated by 10% SDS-PAGE, transferred to a nitrocellulose membrane and probed with phospho-p38 and β-actin antibodies. B) CD14 was knocked down using lentiviral transduction as in Figure 1B & C. MΦ were incubated with *B. burgdorferi* for 30 min and phospho-p38 and β-actin were detected as in Figure 4A. C) CD14^+/+^ MΦ were treated with DMSO, SB202190 (0.5 µM) or arctigenin (1 µM) for 30 min prior to incubation with *B. burgdorferi* and total RNA was analyzed by qPCR for *inos* and *socs3*. D) CD14^+/+^ MΦ were treated as described in (C), and culture supernatants were analyzed for TNF-α by CBA. E) CD14^+/+^ MΦ were treated with DMSO or increasing concentrations of arctigenin or SB202190 for 30 min prior to incubation with *B. burgdorferi*. Cell culture supernatants were collected 24 h p.i. and TNF-α was measured by CBA. F) CD14^+/+^ MΦ were treated with DMSO or increasing concentrations of SB203580 for 30 min prior to incubation with live *B. burgdorferi* or *B. burgdorferi* lysate (10µg/ml). Cell culture supernatants were assayed for TNF-α by CBA. Results represent mean±SEM from two to five independent experiments. *P<0.05, **P<0.01, ***P<0.001.

p38-STAT1-IRF1 signaling positively regulates *socs* activity [26],[30] and negatively regulates *tlr2* activity [37]. To determine whether CD14 signaling modulates this axis, the phosphorylation state of p38 was evaluated in CD14^−/−^ MΦ. In contrast to cells expressing CD14, detectable levels of phospho-p38 were observed only transiently in *B. burgdorferi*-stimulated CD14^−/−^ MΦ (Figure 4A). This result was confirmed and extended using the phospho-specific Cytometric Bead Array (CBA) assay which showed that *B. burgdorferi* stimulated higher phospho-p38, but not phospho-Erk or Jnk, levels in CD14^+/+^ than in CD14^−/−^ MΦ (data not shown). The dependence of p38 activation on CD14 was confirmed using the shRNA targeting strategy described above. As seen in Figure 4B, CD14 shRNA, but not a scrambled shRNA control, dampened the *B. burgdorferi*-induced phosphorylation of p38.

Pharmacological inhibition of p38 has implicated this molecule in proinflammatory responses to a variety of microbial, host, and environmental stimuli [20]. Thus, the coexistence of higher levels of phospho-p38 with lower cytokine production by *B. burgdorferi*-stimulated CD14^+/+^ MΦ is contrary to much of the published literature. To clarify the role of p38 in our model, arctigenin (a general MAPK inhibitor) and SB202190 (a p38-specific inhibitor) were used. Both inhibitors significantly lowered the transcription of *inos* and *socs3* in response to *B. burgdorferi* (Figure 4C), thereby relieving the SOCS-mediated inhibition of TNF-α release by wild-type cells (Figure 4D), an effect that was dose-dependent (Figure 4E). Enhanced TNF-α release also is observed with another p38-specific inhibitor, SB203580, but not when cells were co-incubated with a borrelial lysate (10 µg/ml) (Figure 4F). This latter finding is reflective of the widely-reported observation that p38 inhibition ablates cellular inflammatory responses to isolated bacterial DAMPs [22], [38]–[41] and underscores the difference between stimulation of cells with live spirochetes as opposed to spirochetal lysates or lipoproteins [10],[22],[42],[43].

### CD14 deficiency alters PI3K recruitment to the lipid raft, AKT activity, and downstream p38 signaling

**Figure 5:**
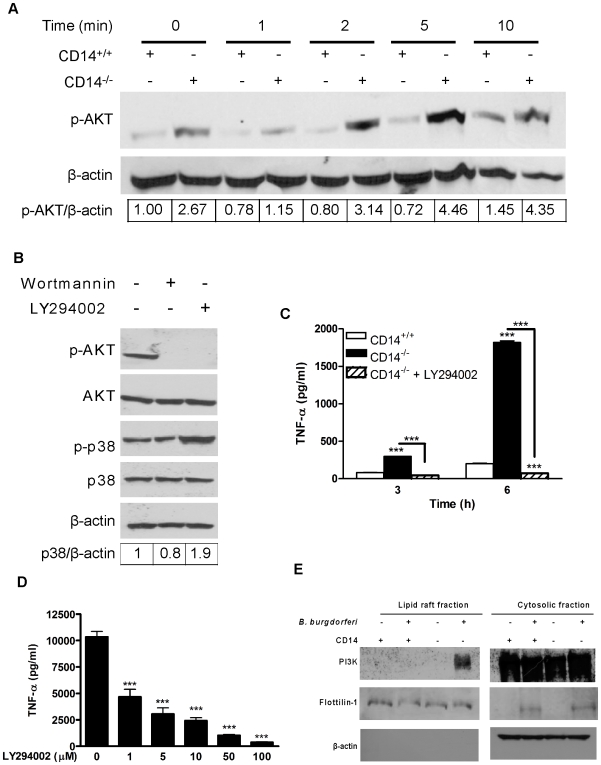
Inhibition of *B. burgdorferi*-induced AKT activation in CD14^−/−^ MΦ reestablishes p38 activity and restores negative regulation of cytokine production. A) Equal protein from lysed CD14^+/+^ and CD14^−/−^ MΦ incubated with *B. burgdorferi* were separated by SDS-PAGE, transferred to a nitrocellulose membrane and probed for phospho-AKT and β-actin. B) CD14^−/−^ MΦ were treated with DMSO or PI3K inhibitors [wortmannin (100nM) or Ly294002 (100µM)] for 30 min prior to incubation with *B. burgdorferi*. Western blots were probed with phospho-specific AKT and p38 antibodies, then striped and reprobed for total AKT, p38 and β-actin. C) CD14^−/−^ MΦ were treated with Ly294002 (100µM) for 30 min prior to incubation with *B. burgdorferi* for 3 and 6 h. Culture supernatants were assayed for TNF-α by CBA. TNF-α release by stimulated CD14^+/+^ MΦ served as a control. D) CD14^−/−^ MΦ were treated with DMSO or LY294002 for 30 min prior to incubation with *B. burgdorferi*. Culture supernatants were collected 24 h p.i. and TNF-α was measured by CBA. E) Lipid rafts were isolated from CD14^+/+^ and CD14^−/−^ MΦ incubated with or without *B. burgdorferi* at a MOI of 10 for 5 min. An equal volume of lipid raft and cytosolic fractions were separated by 10% SDS-PAGE, transferred to a nitrocellulose membrane and probed with antibodies directed against PI3K, flotillin-1, and β-actin. Results represent mean±SEM from two to five independent experiments. ***P<0.001.

p38 is negatively regulated by AKT, a serine-threonine kinase activated via PI3K [44],[45]. To determine whether the diminished p38 activity in CD14^−/−^ MΦ simply reflected AKT function, the levels of phospho-AKT were measured in *B. burgdorferi*-activated MΦ. The phospho-AKT pool in CD14^−/−^ MΦ was significantly increased above baseline and beyond that seen in cells expressing CD14 (Figure 5A). Of note, peak phosphorylation of AKT in CD14^−/−^ cells (seen at 5 min) immediately preceded the disappearance of phospho-p38 (Figure 4A, 10 min) suggesting a strict regulatory network. The PI3K inhibitors wortmannin and Ly294002 were used to determine whether elevated AKT activity was responsible for the decreased phospho-p38 levels in CD14^−/−^ MΦ. As shown in Figure 5B, exposure of cells to either inhibitor reduced the phospho-AKT pool. Blocking PI3K-dependent AKT function via Ly294002, but not wortmannin, resulted in a ∼2-fold increase in the phospho-p38 pool following co-incubation with *B. burgdorferi* (Figure 5B). The inability of wortmannin to have the same effect as Ly294002 may be attributable to its known “off target” inhibition of the MAPK pathway [46]. To explore further how signaling through the PI3K/AKT axis influences *B. burgdorferi*-induced CD14^−/−^ MΦ activation, spirochetes were incubated with cells that were untreated or treated with Ly294002. Enhanced phosphorylation of p38 (Figure 5B) correlated with a profound reduction in *B. burgdorferi*-induced TNF-α secretion (Figure 5C), an effect that was dose-dependent (Figure 5D). Similar results were obtained with the use of another PI3K inhibitor, PI-103 [47], (data not shown). Perturbation of the PI3K/AKT/p38 axis in CD14^−/−^ MΦ reduced cytokine production to the level observed in CD14^+/+^ cells (Figure 5C).

PI3K translocates from the cytosol to the inner leaflet of the plasma membrane (*i.e.*, cytoplasmic face of the lipid raft) upon activation of MΦ by various DAMPs [48]. Given the capacity of PI3K to phosphorylate AKT we characterized the composition of the lipid raft formed during the interaction of *B. burgdorferi* with CD14^+/+^ and CD14^−/−^ MΦ to uncover the mechanism(s) whereby the phospho-AKT pool is greater in cells lacking CD14. Lipid raft and non-raft fractions were isolated from cells incubated with or without *B. burgdorferi* for 5 min. The lipid raft and cytosolic fractions were probed for PI3K, flotillin-1 (a lipid raft marker), and actin (a cytosolic marker). The only lipid raft fraction to contain significant amounts of PI3K was from the CD14^−/−^ MΦ incubated with *B. burgdorferi* (Figure 5E). Some flotillin-1 was present in the non-raft fraction of both cell types following incubation with *B. burgdorferi* and likely is due to tethering of the lipid raft to the actin cytoskeleton during phagocytosis [49], which would inhibit the ability of those rafts to float on a sucrose gradient. These data suggest that regulated localization of PI3K (and likely other molecules) to *B. burgdorferi*-induced lipid rafts links proximal membrane events to distal intracellular signaling cascades.

### In the absence of CD14, *B. burgdorferi*-stimulated cytokine production is both dependent and independent of TLR2 and PI3K signaling

**Figure 6:**
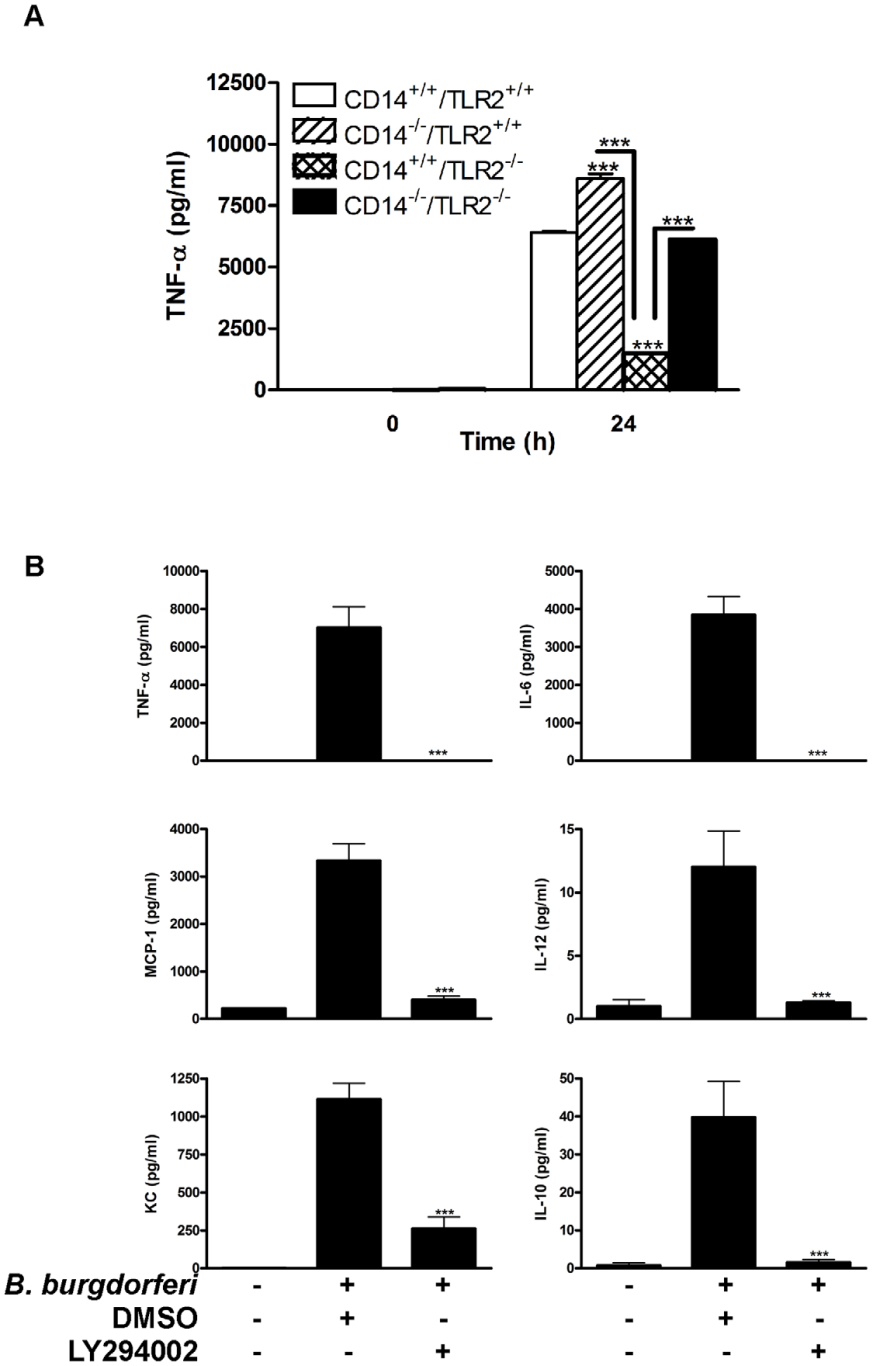
TLR2 plays a partial role in both CD14-dependent and -independent cytokine production. A) MΦ isolated from CD14^+/+^, CD14^−/−^, TLR2^−/−^ and CD14^−/−^/TLR2^−/−^ mice were incubated with *B. burgdorferi* and culture supernatants were assayed for TNF-α by CBA. B) *B. burgdorferi* was incubated with MΦ isolated from CD14^−/−^/TLR2^−/−^ mice in the presence of DMSO or 100µM Ly294002 for 24 h and cytokines were measured by CBA. Results represent mean±SEM from three independent experiments. ***P<0.001.

Coordinate signaling through CD14 and TLR2 activates PI3K, NF-κB, and p38-MAPK pathways [2],[50] to orchestrate both the initiation and resolution of inflammatory responses to *B. burgdorferi*. CD14 deficiency alters PI3K localization to lipid rafts and excessive phosphorylation of AKT along with higher transcription and surface expression of TLR2 is the result. Since TLR2 signaling regulates PI3K activity and leads to AKT activation [2], we asked whether higher TNF-α production by *B. burgdorferi*-stimulated CD14^−/−^ MΦ depends upon TLR2-mediated activation of AKT and/or engagement of another non-TLR2 receptor(s). To better appreciate the individual and combined contribution of CD14 and TLR2 signaling to the MΦ response to *B. burgdorferi*, mice deficient for CD14 or TLR2 (each backcrossed 10 generations onto a C3H/HeN background) were crossed to establish animals that are homozygous recessive for both loci. MΦ expressing CD14 and TLR2 and those deficient for individual or both loci were co-incubated with spirochetes and TNF-α levels were measured (Figure 6A). In the absence of CD14, TNF-α production was significantly higher compared to their wild-type counterparts at 24 h p.i.. TLR2 deficiency had the opposite effect insofar as release of TNF-α (Figure 6A) and other cytokines (data not shown) was significantly reduced compared to wild-type MΦ. Despite this reduction, cytokine release by TLR2^−/−^ MΦ was greater than that observed with unstimulated control cells (*P*<0.01), implicating the existence of a TLR2-independent pathway, one that may or may not be triggered by non-lipoprotein spirochetal constituents. Interestingly, the cell's capacity to secrete TNF-α in the absence of both TLR2 and CD14 was partially or fully restored to the levels released by CD14^−/−^ and wild-type MΦ, respectively. Restoration of TNF-α production by CD14^−/−^/TLR2^−/−^ MΦ further supports the notion that *B. burgdorferi*-induced signaling is not solely dependent upon TLR2 signaling, nor is CD14 signaling as demonstrated by the phenotypic difference in cytokine response observed between TLR2^−/−^ MΦ and CD14^−/−^/TLR2^−/−^ MΦ (Figure 6A). These findings raise the intriguing question of what ultimately controls cytokine production in the absence of both the CD14 and TLR2 pathways. As seen in Figure 6B, production of a variety of cytokines by CD14^−/−^/TLR2^−/−^ MΦ is ablated in the absence of PI3K activity. This result suggests that both TLR2-dependent and -independent activation of PI3K contributes to the hyper-inflammatory state in CD14^−/−^ MΦ induced by *B. burgdorferi*. More broadly, it also demonstrates that, notwithstanding similarities in the exaggerated Lyme disease phenotype observed in CD14^−/−^ and TLR2^−/−^ mice [51],[52], these two signaling pathways are not entirely synonymous.

### CD14 licenses MΦ to become tolerant to perpetual bacterial stimulation

**Figure 7:**
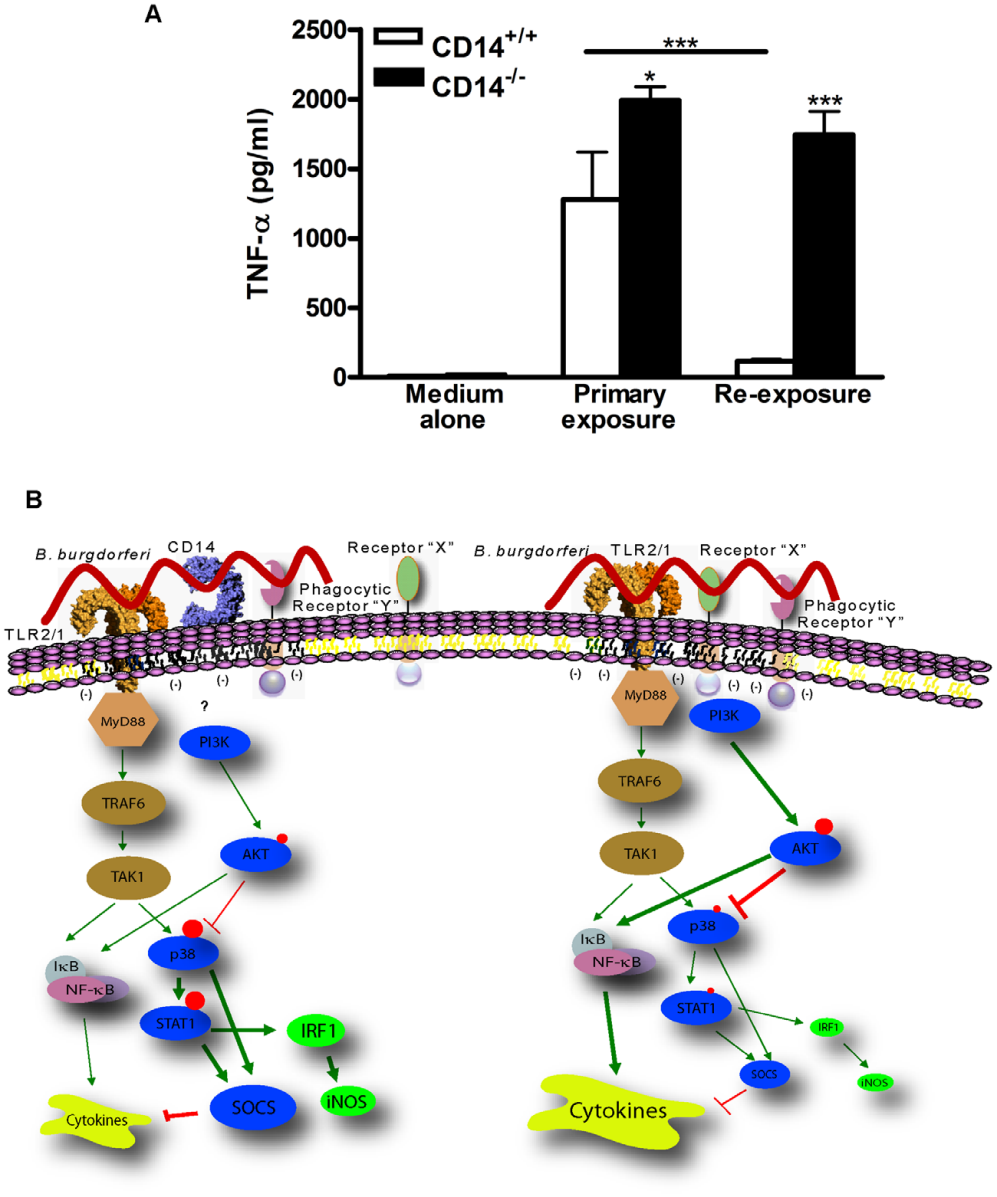
CD14-dependent signaling is a requirement for *B. burgdorferi*-induced tolerance in MΦ. A) CD14^+/+^ and CD14^−/−^ MΦ were incubated with *B. burgdorferi* at a MOI of 10 for 12 h (primary exposure). Cell monolayers were washed extensively followed by reexposure (secondary exposure) to medium containing *B. burgdorferi* for an additional 6 h. The TNF-α level in culture supernatants from cells rexposed to spirochetes was compared with levels released by MΦ which received only a primary exposure to *B. burgdorferi*. B) A schematic diagram illustrating CD14-dependent and -independent signaling events in response to *B. burgdorferi*. In the absence of CD14 neither binding nor uptake of *B. burgdorferi* via a putative Phagocytic Receptor “Y” is affected. However, localization of PI3K to the signaling platform is altered, perhaps owing to the presence of Receptor “X”, which increases the pool of phospho-AKT resulting in only transient p38 activity. Impaired p38 activity correlates with increased transcription and expression of TLR2, reduced activation of STAT1 and IRF1, and decreased induction of SOCS, all of which are necessary to modulate the intensity and duration of Lyme borreliosis. Ultimately, it is CD14-dependent induction of p38-mediated SOCS activity which regulates the intensity and duration of macrophage response to *B. burgdorferi* through the induction of tolerance. The size of the red circles indicates the degree of phosphorylation of the indicated molecule and the size of the individual molecules is reflective of relative transcript/protein levels. The thickness of lines in the model reflects the intensity of the positive or negative action on downstream molecules.

TLR activation of MΦ concomitantly triggers cytokine production and its negative regulation to temper the extent and duration of inflammation thereby mitigating immunopathology. Given the role of p38 in the induction of tolerance [24] and the observation that *B. burgdorferi* induces lower p38 activity in CD14^−/−^ MΦ, we asked whether these cells fail to become tolerant to perpetual stimulation by spirochetes. To address this question, CD14^+/+^ and CD14^−/−^ MΦ were incubated with medium alone or *B. burgdorferi* at a MOI of 10 for 12 h. Afterwards, cells were extensively washed and reexposed to *B. burgdorferi* at a MOI of 10 for an additional 6 h (Figure 7A). Upon primary exposure, cells lacking CD14 secreted significantly more TNF-α than their wild-type counterparts (*P*<0.05). Also as anticipated, CD14^+/+^ MΦ receiving a primary exposure (non-tolerized) released significantly more cytokine than cells which were reexposed to spirochetes (tolerized) (*P*<0.001). In contrast, the amount of TNF-α produced by CD14^−/−^ MΦ receiving a primary exposure to *B. burgdorferi* was identical to that released by cells after reexposure and significantly greater than that released by tolerized CD14^+/+^ cells (*P*<0.001). These data support a novel and indispensable role for CD14-dependent p38 regulation of MΦ function through the induction of tolerance to natural bacterial infection.

## Discussion

Coordinate signaling through CD14 and TLR2 orchestrates both the initiation and resolution of inflammatory responses to *B. burgdorferi*. During natural infection, initiation of the host response begins with CD14 recognition of borrelial lipoproteins and subsequent activation of TLR2 [10],[52],[53]. As described herein and elsewhere [10], the absence of CD14 results in greater bacterial burden, more severe histopathology, and dysregulated production of a variety of immunomodulators. Contrary to current thinking, we demonstrate that CD14 signaling is dispensable for the induction of inflammation and, instead, tempers the intensity (*e.g.*, cytokine levels) and duration (*e.g.*, joint swelling) of innate inflammatory responses to *B. burgdorferi*. In the face of continued exposure to stimuli of bacterial origin this inflammatory “temperance” reflects negative regulation of TLR signaling which tolerizes MΦ via the action of SOCS. In turn, SOCS activity is modulated via the PI3K/AKT/p38-MAPK pathway. The initiation of these signaling events occurs at the contact surface between the spirochete membrane and that of the phagocyte (Figure 7B).

Despite the fact they comprise only 0.5% of eukaryotic cellular proteins, GPI-anchored polypeptides (*e.g.*, CD14) have diverse roles in cellular physiology - from contributing to cell development and viability to host cell defense against microbial insults [54]. Due to the hydrophobic nature of GPI-anchors such proteins constitutively reside in cholesterol-rich detergent-insoluble/detergent-resistant membranes [55],[56]. These lipid rafts serve as signaling platforms for various receptors, the best example of which is the T-cell receptor (TCR). Lacking an extended cytoplasmic tail, the TCR resides outside lipid rafts until engaged through recognition of MHC-peptide complex displayed on antigen presenting cells [57]. Upon ligand binding, the TCR is recruited to the lipid raft where it associates with kinase signaling components such as Lck and Lat. With TCR engagement there is a concomitant exclusion of the phosphatase CD45 from the lipid raft to prevent inactivation of kinases in the signaling platform [57]. Accumulating evidence suggests that lipid rafts also are important in initial bacterial and viral recognition and internalization of pathogens by the MΦ [58]. Depending upon the ligand, different receptor components, adaptor proteins, and kinases/phosphatases coalesce within lipid rafts along with CD14 and TLRs to orchestrate cellular responses to DAMPs [59]. Engagement of receptors residing within the lipid raft alters the surface potential of the inner leaflet of the plasma membrane thus regulating recruitment of non-receptor dependent signaling molecules (*e.g.*, PI3K) [60],[61]. We found that following activation by *B. burgdorferi*, significantly more PI3K is recruited to the lipid rafts of MΦ which lack CD14 than those expressing CD14. Thus, PI3K appears to link the spirochete-MΦ plasma membrane “interaction” platform with the AKT/p38-MAPK pathway within the cytosol. Perhaps analogous to the partitioning of CD45 during TCR signaling, it is intriguing to speculate whether a specific protein(s) is excluded from and/or recruited to the lipid raft depending on whether CD14 is present or absent. Such a protein(s) is represented as “Receptor X” in our model (Figure 7B). In the former case exclusion of a protein(s) may eliminate a necessary docking partner for PI3K (*e.g.*, Src kinases/c-Kit, [48]) and in the latter case, a protein(s) might actively prohibit entry of PI3K into a CD14-containing lipid raft. Alternatively, differential distribution of lipids may distinguish the initiation of signaling within CD14-containing and -deficient lipid rafts [62]. As an example, receptors are differentially recruited into ceramide- versus cholesterol-rich lipid rafts [63].

Upon binding of pathogens to the phagocyte surface, receptor-dependent cytoskeletal rearrangements occur via recruitment of actin-related proteins to the lipid raft, a necessary prelude to internalization of *B. burgdorferi* [64]. A variety of molecules have been implicated in the process of binding and phagocytosis of spirochetes including members of the integrin family such as α_3_β_1_, α_4_β_3_, and CD11b/CD18 [65]–[70], and the cytosolic adapter protein MyD88 [64],[71],[72]. A report that CD14 acts as a unique phagocytic receptor for Gram-negative bacteria [73], suggested that this molecule might serve a similar function in spirochetal uptake. However, we found that CD14^+/+^ and CD14^−/−^ MΦ were equally adept at binding and internalizing *B. burgdorferi*. As such, a Phagocytic Receptor “Y” (Figure 7B) of unknown identity acts in tandem with/without CD14 to concomitantly initiate spirochetal uptake and innate immune signaling in a TLR2-dependent and -independent fashion. Importantly, live spirochetes versus lysates appear to exhibit a differential capacity to engage Phagocytic Receptor “Y” which underlies why equivalent amounts of each stimulus elicit different amounts of cytokine [10],[22],[42],[43], and perhaps why p38 plays a dichotomous role in regulating the TNF-α production described herein. Despite similar spirochetal uptake, greater recovery of bacteria from infected CD14^−/−^ MΦ and mice suggested differential killing of *B. burgdorferi* in the presence or absence of CD14. Bacterial killing within MΦ occurs by two principal means: i) the generation of RNS/ROS which reduces phagosomal pH thus facilitating their fusion with lysosomes and ii) the production of antimicrobial peptides [35]. Several recent *in vitro* studies demonstrate the susceptibility of *B. burgdorferi* to RNS/ROS [74]–[77]. Additionally, *in vivo* studies have shown that RNAi suppression of tick salivary proteins capable of neutralizing RNS/ROS at the vector-host-pathogen interface increase the survival of spirochetes in skin and facilitate their dissemination throughout the host [78]–[80]. Consistent with this latter finding the higher bacterial burden observed in CD14^−/−^ MΦ and mice is associated with lower *in vitro* and *in vivo* transcription of *inos*. Release of antimicrobial peptides by human MΦ is triggered by p38-dependent vitamin D_3_ receptor (VDR) signaling [81]–[85]; however, whether the same regulatory network exists in mouse MΦ is unclear. Interestingly, transcription of *vdr* was dramatically lower in CD14^−/−^ MΦ than in their wild type counterparts (data not shown). Thus differential transcription of *inos* and *vdr* may explain, at least in part, the impaired ability of CD14^−/−^ mice to clear spirochetes from infected organs. Differential transcription of *inos*, *vdr*, *socs*, and phosphorylation of STAT1 suggest the involvement of p38-MAPK, which controls all these events in MΦ.

AKT has pleiotropic effects that include promotion of cell survival, NF-κB activation, and inhibition of ASK1, a MAPKKK responsible for p38 activity [44],[45]. p38 activity is associated with NF-κB activation and stabilization of mRNA encoding proinflammatory cytokines such as TNF-α [23]. The observation that inhibition of PI3K prevented phosphorylation of AKT and thus increased p38 activity in *B. burgdorferi*-activated CD14^−/−^ MΦ is consistent with the inverse relationship between AKT and p38 activity [44],[45]. Unexpectedly, PI3K inhibition also resulted in decreased TNF-α production, a finding inconsistent with higher p38 activity and its “accepted” role as a proinflammatory mediator [20]. Nevertheless, also consistent with our finding, direct inhibition of p38 in *B. burgdorferi*-stimulated CD14^+/+^ cells with either general or specific antagonists resulted in lower *inos* and *socs3* transcription and a dose-dependent increase in TNF-α. This provocative finding was further supported by the ability of shRNA to suppress CD14 expression in MΦ thereby reducing phosphorylation of p38 and increasing TNF-α release in response to spirochetes. Finally, in a murine model of Lyme borreliosis transient inhibition of p38 through use of inhibitors was found to increase *B. burgdorferi*-induced carditis and is associated with a higher bacterial burden in infected tissues [personal communication; Dr. Juan Anguita, Ph.D., (Veterinary & Animal Sciences, University of Massachusetts, Amherst)]. This altered disease phenotype is reminiscent of the increased histopathology and higher bacterial burden in the infected tissues of CD14^−/−^ mice whose MΦ only transiently produce active p38 following activation by *B. burgdorferi*.

Contrarily, p38 inhibition reduces TNF-α secretion by the mouse RAW264.7MΦ cell line in response to borrelial lysates [22]. We too report that inhibition of p38 in CD14^+/+^ MΦ reduced TNF-α production in response to borrelial lysates, but not live spirochetes, and did so in a dose-dependent fashion. One potential explanation for the contradictory finding that p38 inhibition augments the inflammatory response to live bacteria and suppresses the response to lysates is that the context in which bacterial DAMPs are recognized influences the downstream signaling cascades initiated within the host cell. Supportive of this idea, several studies have demonstrated that live versus lysed spirochetes (wherein molar equivalency of the two stimuli is established) elicits a differential cytokine response from MΦ of mouse and human origin [10],[42],[43],[86]. The anti-inflammatory capacity of p38 is further demonstrated in mouse models of pneumococcal pneumonia and tuberculosis where its inhibition results in impaired bacterial clearance and increased TNF-α production both *in vitro* and *in vivo* [87]. It also has been established that p38 induces the shedding of TNF receptor-1 from activated cells thus dampening their responsiveness to TNF-α [88]. The combination of increased TNF-α production and maintenance of its cognate receptor on the cell surface likely contributes to the cytokine “surge” and exacerbates the pathology associated with bacterial infection. Taken together, these results challenge the notion that disaggregated or isolated DAMPs (*e.g.*, lysates, purified LPS, lipoproteins, *etc.*) are equivalent to the whole organisms from which they are generated [10],[24]. They also sound a cautionary note regarding application of p38 inhibitors to treat inflammatory disorders, especially those of infectious origin, wherein blockade of this signaling pathway may exacerbate rather than ablate inflammation. Development of immunotherapeutic strategies that enhance the action of SOCS may represent a more fruitful avenue in pursuit of novel anti-inflammatory drugs [31], [89]–[91]. Despite the concordance of results described above it also must be acknowledged that commonly used inhibitors of PI3K and p38 demonstrate off-target effects including blockade of other kinases and phagocytosis when used at high concentrations. In the present study, the use of inhibitors at low concentrations partially mitigates the likelihood of such off-target effects, however, left unclear is their potential impact on phagocytosis of *B. burgdorferi* and subsequent cytokine release by host cells. Further complicating this issue is the existence of eight isoforms of PI3K and four isoforms of p38. The relative contribution of different isoforms to activation of host cells and the influence of exposure to live versus lysed spirochetes on signaling are currently under investigation employing a shRNA strategy that will obviate concerns raised by non-specificity.

A correlative relationship exists between phagocytosis of bacteria and cytokine release [92]. For example, MyD88 deficiency impairs both uptake of *B. burgdorferi* by mouse MΦ and subsequent transcription of *tnfα* and *il6* [72]. Under other circumstances (*i.e.*, TLR2 deficiency), while uptake of spirochetes by MΦ was unaffected, the transcription of a variety of cytokine and chemokine genes was significantly reduced [72]. Similarly, we found that CD14^+/+^ and CD14^−/−^ MΦ were equally adept at binding and internalizing *B. burgdorferi* and cytokine secretion was altered; however, in this case, the proinflammatory response of MΦ lacking CD14 was significantly greater than that seen with their wild-type counterparts. Differences in TNF-α levels might simply reflect the greater survival/persistence of spirochetes within and expression of TLR2 by CD14^−/−^ versus CD14^+/+^ MΦ, either or both of which could potentiate cytokine secretion. However, evidence provided by *in vivo* experiments argues against cytokine secretion being directly linked to spirochetal burden insofar as higher cytokine levels in the serum of CD14^−/−^ mice at 1 and 3 week p.i. (Figure S2) precedes the significant differences in bacterial burden observed at 6 weeks (Table S1). We propose that the aforementioned response is more likely a reflection of the inability of *B. burgdorferi* to induce tolerance in MΦ lacking CD14 which, in turn, results from impaired p38-mediated SOCS activity. Trace amounts of DAMPs can impose tolerance on MΦ thus making them less or unresponsive following subsequent reexposure to stimuli of an identical or similar (cross-tolerization) nature [93],[94]. Relevant to the phenomenon of tolerance, Dennis *et al.* reported that recognition of *B. burgdorferi* activates SOCS [95] and Diterich *et al.* showed that *B. burgdorferi* can tolerize MΦ [96] and induction of tolerance requires p38-mediated stimulation of SOCS [24], [28], [97]–[101]. Here we mechanistically link these previous findings by showing that CD14 recognition of *B. burgdorferi* triggers p38-dependent SOCS activity to impose a tolerant phenotype within MΦ. Interestingly, this CD14-dependent mechanism of tolerance may be operative in the absence of TLR2 as well, a notion supported by the observation that TLR2^−/−^ MΦ produce significantly less cytokine than when these cells also are deficient for CD14. Uncontrolled release of proinflammatory cytokines by cells lacking CD14 likely is further exacerbated by their diminished production of the anti-inflammatory cytokine IL-10, which normally would temper such responses (data not shown). Thus, activation of MΦ in the absence of CD14 may represent the “perfect storm” of increased bacterial burden and expression of TLR2 and reduced tolerance to perpetual stimulation by *B. burgdorferi*. It is this dysregulated response at the cellular level which underpins the altered Lyme disease phenotype observed in CD14^−/−^ mice.

*In toto*, we detail a critical and unanticipated role for CD14 in downmodulating TLR2-dependent and -independent pathways which regulate NF-κB signaling events. We provide evidence that reduced SOCS activity licenses greater cytokine production through diminished negative regulation of the TLR2 pathway. CD14 exerts its influence on the intensity and duration of inflammation through the PI3K/AKT/p38-MAPK axis which serves as a “rheostat” to finely modulate NF-κB activity, a critical regulator of the host's inflammatory response to pathogenic challenge. An intriguing question raised by these findings is whether individual differences in the clinical course of infection might reflect the susceptibility of the patient's innate immune system to tolerization.

## Methods

### Reagents

All buffers and reagents were prepared to minimize contamination with environmental LPS by utilizing baked (180°C for 4 h) and autoclaved glassware, disposable plasticware, and pyrogen-free H_2_O.

### Cultivation of *B. burgdorferi*

Low-passage *B. burgdorferi* strain 297 were maintained at 23°C in Barbour-Stoenner-Kelley medium containing 6% normal rabbit serum (BSK_complete_) from Pel-Freez Biologicals (Rogers, AR) and then temperature-shifted to 37°C. Increased expression of OspC was confirmed by silver staining of whole borrelial lysates separated by SDS-PAGE. *B. burgdorferi* 297 expressing green fluorescent protein were cultivated in BSK_complete_ containing 400 µg/ml kanamycin and were grown at 37°C until mid- to late-logarithmic phase for subsequent use in phagocytosis experiments as previously described [102]. For the generation of borrelial lysates temperature shifted bacteria were harvested by centrifugation at 8000×*g*, washed twice with PBS lacking Ca^++^ and Mg^++^, and finally resuspended in PBS. Lysates were prepared by sonicating the bacteria for 30 s (with three separate 10 s pulses at 30 s intervals) using a 60-Dismembrator (Fisher Scientific, Waltham, MA). The total protein content of lysates was determined using the BCA method.

### Mice and infection protocol

Four to eight week-old C3H/HeN (CD14^+/+^) mice (Taconic, Germantown, NY) were housed in the Animal Resources Facility at Albany Medical College. Food and water were provided *ad libitum* and all animal procedures were approved by the Institutional Animal Care and Use Committee of Albany Medical College. CD14^−/−^ mice were generated as previously described [6] and subsequently backcrossed 10 generations onto a C3H/HeN background [10]. TLR2^−/−^ mice were provided by Tularik Inc. (now Amgen Inc., South San Francisco, CA), generated by Deltagen Inc. (Menlo Park, CA) [103] and were backcrossed 10 generations onto a C3H/HeN background. C3H/HeN mice deficient for CD14 and TLR2 were crossed to establish animals that are homozygous recessive for both alleles.

Mice were infected via intradermal administration of 1×10^5^ spirochetes over the sternum. For some experiments mice were infected using *Ixodes scapularis* ticks carrying *B. burgdorferi* as previously described [10]. At one week intervals, tibiotarsal joint thickness was measured using digital calipers and bacterial burden in infected tissues was determined using isolated genomic DNA as previously described [10]. Total RNA also was isolated from infected tissues for qPCR as described below.

### Isolation and differentiation of MΦ

MΦ were isolated from the bone marrow of six to eight week-old mice. Briefly, bone marrow cells recovered by flushing femurs and tibia with DMEM were incubated in tissue culture-treated 25cm^2^-flasks (BD Falcon, BD Biosciences, San Jose, CA) overnight at 37°C with 5% CO_2_ to eliminate adherent fibroblasts, granulocytes, and any contaminating MΦ [104]. The following day, 1×10^7^ suspension cells were maintained in 10-cm^2^ bacteriological Petri dishes (BD-Falcon) for three days with DMEM supplemented with 10% fetal bovine serum, 20% L292-cell conditioned media, 0.01% HEPES, 0.01% sodium pyruvate, and 0.01% L-glutamine. Cultures were supplemented with 5 ml of the above-described medium and seven days after isolation cell monolayers were recovered using ice-cold PBS and scraping. Single cell suspensions were used immediately or frozen in liquid nitrogen with 20% FBS and 10% DMSO for use in future experiments.

### *B. burgdorferi*-MΦ co-incubation

MΦ were seeded into 6-well tissue culture-treated plates at a concentration of 1×10^6^ cells/2 ml/well and allowed to adhere overnight. The following day, *B. burgdorferi* were enumerated and resuspended as described above. MΦ were washed twice with serum-free DMEM to remove any traces of FBS and spirochetes (resuspended in DMEM + 4% autologous serum) were added at a MOI of 10 and co-incubated for different time intervals at 37°C in 5% CO_2_. Cells incubated with DMEM + 4% autologous serum alone served as mock-infected controls. For tolerance experiments, MΦ were initially incubated with *B. burgdorferi* at a MOI of 10 for 12 h at 37°C in 5% CO_2_. Supernatants were collected to determine TNF-α levels by CBA and cells were washed extensively with pre-warmed serum-free medium before addition of fresh medium containing *B. burgdorferi* at the same concentration as used for the primary exposure. Supernatants were collected after an additional 6 h of co-culture and TNF-α levels were again measured.

### Quantitative real-time PCR

Total RNA was isolated from MΦ using the RNeasy Mini Kit (Qiagen GmbH, Hilden, Germany) as per the manufacturer. The amount and purity of RNA was quantified by Biophotometer (Eppendorf AG, Hamburg, Germany) and 0.5µg were used for reverse transcription of cDNA using Superscript II (Invitrogen Corporation, Carlsbad, CA). cDNA (20µl) served as the template in quantitative real-time PCR (qPCR) analysis using Mouse TLR Signaling Pathway RT^2^ Profiler™ PCR Arrays (SABiosciences, Frederick, MD). This qPCR methodology directly quantifies transcript levels based upon the 2^−ΔΔCt^ method through measurement of SYBR green fluorescence using an iQ5 real-time PCR detection system (Bio-Rad Laboratories, Hercules, CA).

For transcriptional analysis of genes not represented on the RT^2^ Profiler™ PCR Array, unique genes were interrogated by qPCR in a final volume of 25 µl containing: 12.5 µl of 2× SYBR green master mix (Bio-Rad Laboratories), 25 pico-moles of forward and reverse primers, and 0.2 µl of cDNA. Primers were designed using Beacon Designer version 7.0 software (PREMIER Biosoft Intl, Palo Alto, CA) and the sequences of specific primer sets are provided in Table S2. Amplification conditions were 95°C (3 min) and 40 cycles of 95°C (15 s), 55°C (40 s) and 72°C (30 s). All the qPCR reactions were run in triplicate with no-template controls (NTC) and mean c*T* values were used for all the calculations using 18S rRNA as an internal normalization control. Transcript levels for infected groups are presented as a fold change over their corresponding uninfected control group. A greater than two-fold change with respect to mock control was considered significant.

### Western blot analysis

Antibodies directed against SOCS1, SOCS3 and CIS were obtained from Santa Cruz Biotechnology, Inc. (Santa Cruz, CA), antibodies against AKT, STAT1, STAT3 and p38 were obtained from Cell Signaling Technology, Inc. (Danvers, MA), and antibodies against β-actin were obtained from Bethyl Laboratories, Inc. (Montgomery, TX). Protein samples (25–100µg, depending on the target) were resolved by SDS-PAGE and transferred to nitrocellulose using semi-dry transblot (Bio-Rad Laboratories, Hercules, CA). The membrane was blocked with 5% non-fat milk and then incubated overnight at 4°C with primary antibody (1∶1000 for AKT and STATs, 1∶100 for SOCS, and 1∶10,000 for β-actin). Membranes were probed with HRP-conjugated anti-rabbit IgG (Cell Signaling Technology) diluted 1∶2000. Specific signal was developed using the SuperSignal West-Dura chemiluminescent substrate (Pierce Endogen, Rockford, IL).

### Cytometric bead array (CBA) for cytokine and phospho-protein analysis

Cytokine levels were measured in recovered culture supernatant using the Mouse Inflammation CBA kit and a FACSArray flow cytometer [BD Immunocytometry Systems (BDIS), San Jose, CA]. Data was acquired and analyzed using BD FACSArray software and FCAP Array software, version 1.0 (BDIS), respectively. For phospho-protein analysis, the protein content of the samples was normalized, and samples were analyzed using a phospho-specific CBA kit.

### Determination of recoverable live *B. burgdorferi* from MΦ

To enumerate *B. burgdorferi* recovered from CD14^+/+^ and CD14^−/−^ MΦ we used a variation on the classical tissue culture infective dose (TCID) method used in virology to determine the viral titer necessary to infect 50% of target cells. Briefly, 5×10^4^ CD14^+/+^ and CD14^−/−^ MΦ were co-incubated for 6 h with *B. burgdorferi* at a MOI of 10. The MΦ were washed with PBS lacking Ca^++^ and Mg^++^ and the cells were harvested by scraping. Ten-fold serial dilutions were made in BSK_complete_ media down to 10^−7^. Aliquots of 100µl of each dilution were placed in triplicate wells of a 96-well microtiter plate and incubated for 7 days. On the 7^th^ day dark field microscopy was used to visualize spirochetes and bacterial titers were calculated using the Reed-Muench formula [105].

### Inhibition of signaling cascades by pharmacological inhibitors

MΦ were treated with the inhibitors arctigenin (1µM), SB202190 (0.5µM), SB203580 (10µM), wortmannin (100nM), and Ly294002 (100µM) for 30 min prior to addition of *B. burgdorferi* at a MOI of 10. In some experiments a range of inhibitor concentrations was used. DMSO alone served as a control. Culture supernatants were collected for cytokine measurement and cells were lysed for RNA isolation and Western blot analysis.

### Lipid raft isolation

MΦ (1×10^7^) from CD14^+/+^ and CD14^−/−^ mice were lysed after 5 min exposure to *B. burgdorferi* at a MOI of 10 using ice-cold TNE buffer [10mM Tris (pH 7.5), 150mM NaCl and 5mM EDTA] containing 1% Triton X-100. Lysates were centrifuged at 48,000×*g* for 4 h on a sucrose gradient to float the lipid raft fraction from the non-raft fraction. Fractions (40 µl) were resolved by SDS-PAGE and analyzed by Western blotting as described above. Antibodies used at the manufacturer's suggested concentrations included rabbit anti-mouse PI3K (Santa Cruz Biotechnology), flotillin-1 (Cell Signaling Technology), and β-actin (Bethyl Laboratories).

### Two-photon laser confocal microscopy

MΦ were seeded into 8-well chamber slides (Lab-Tek™ II CC2 Chamber Slide™ System) at a concentration of 1×10^5^ cells/200µl/well and allowed to adhere overnight. The following day, after staining the cell membrane using wheat germ agglutinin Alexa Fluor-647, GFP-expressing *B. burgdorferi* were enumerated and added to cell monolayers at a MOI of 100 and co-incubated for different time intervals. The cells were fixed with 1% paraformaldehyde and were mounted in VECTASHIELD® with DAPI (Reactolab SA, Servion, Switzerland). Confocal images were acquired using a Zeiss LSM-510 META-NLO microscope equipped with argon and HeNe lasers. Optically sectioned images were acquired using a 40× objective (512×512 pixel resolution) at 0.5 µm intervals. Image processing and analysis were performed using ImageJ (NIH, v1.41b) and LSM Image Browser (Zeiss, v5.0). Depth coding and green color extraction was used to determine the area representing internalized GFP-labeled *B. burgdorferi*. The hue/saturation was brought up to 100% in Adobe Photoshop and the green color was extracted, imported into ImageJ and the percentage of green area was measured.

### FACS analysis

MΦ (5×10^5^ cells) were aliquoted into 5 ml polystyrene round-bottom tubes (BD Biosciences, Bedford, MA) and incubated with GFP-expressing *B. burgdorferi* resuspended in DMEM+4% autologous serum at a MOI of 10. Cells and bacteria were co-incubated for different time intervals at 4°C or 37°C and then processed for flow cytometric analysis as previously described [10]. Phagocytic indices were calculated using the formula phagocytic index = (% GFP^+^ cells at 37°C×MFI at 37°C)−(% GFP^+^ at 4°C×MFI at 4°C).

### Lentiviral transduction

Knock down of CD14 was achieved using a pRS-shGFP lentiviral expression vector (OriGene Technologies, Rockville, MD) to transduce either a CD14 shRNA or a scrambled control shRNA into CD14^+/+^ MΦ. Briefly, ampho packaging cell lines (Orbigen Inc., San Diego, CA) were transfected with lentiviral vector expressing scrambled shRNA or shRNA targeting CD14 using a standard calcium phosphate method. Cell-culture supernatants were collected 48 h post-transfection and titered virus was added to CD14^+/+^ MΦ in presence of 4µg/ml Polybrene. The efficiency of suppression of CD14 was assessed 72 h post-viral transduction using flow cytometry and Western blot analysis.

### Statistical analysis

A two-way Analysis of Variance (ANOVA) followed by a Fisher LSD post-hoc test (InStat 4.0, GraphPad Software Inc., La Jolla, CA) was used to analyze data presented in Figure 2A. Other data sets were analyzed for statistical significance using a parametric two-way ANOVA with a Bonferroni correction if the data was found to fit a Gaussian distribution (tested using the method Kolmogorov and Smirnov). When not normally distributed the data was analyzed for statistical significance using a non-parametric two-way ANOVA with Mann–Whitney U test. An α = 0.05 level was used to determine whether a significant difference existed between data from CD14^+/+^ versus CD14^−/−^ and untreated versus treated groups.

## Supporting Information

Figure S1 CD14 deficiency undermines the Lyme arthritis-resistant phenotype of C57BL/6 mice. CD14^+/+^ and CD14^−/−^ C57BL/6 mice were infected using *Ixodes scapularis* ticks carrying *B. burgdorferi* and tibiotarsal joint thickness was measured at 1-wk intervals using digital calipers. The horizontal bars indicate mean thickness for each group and the data are representative of two independent experiments (n = 24).
(0.56 MB TIF)

Figure S2 CD14 deficiency results in increased *in vivo* cytokine production in response to *B. burgdorferi*. CD14^+/+^ and CD14^−/−^ C57BL/6 mice were tick-inoculated with *B. burgdorferi* and serum cytokine levels were measured using CBA at 1, 3, and 6 wks p.i.. Results represent mean±SEM from three independent experiments. **P<0.01, ***P<0.001.
(0.22 MB TIF)

Table S1 CD14 deficiency impairs clearance of *B. burgdorferi* in C57BL/6 mice. Mice were sacrificed at 1, 3, and 6 wks post tick-inoculation and DNA was isolated from the indicated tissues for qPCR analysis as described in Figure 2C and Methods. Results represent mean±SEM from three independent experiments (n = 10 to 16) wherein samples were run in triplicate. *P<0.05, **P<0.01.
(0.10 MB PDF)

Table S2 Primer sequences used in qPCR.
(0.10 MB PDF)

Video S1 Movie sequence of stacked optical z section of CD14^+/+^ MΦ shown in Figure 3C.
(7.30 MB MOV)

Video S2 Movie sequence of stacked optical z section of CD14^−/−^ MΦ shown in Figure 3C.
(6.50 MB MOV)
