# Supplementary figures and images for: CD14 Signaling Restrains Chronic Inflammation through Induction of p38-MAPK/SOCS-Dependent Tolerance
Source: PLoS Pathog. 2009 Dec 11;5(12):e1000687. doi: 10.1371/journal.ppat.1000687 (PMC2781632; doi:10.1371/journal.ppat.1000687)

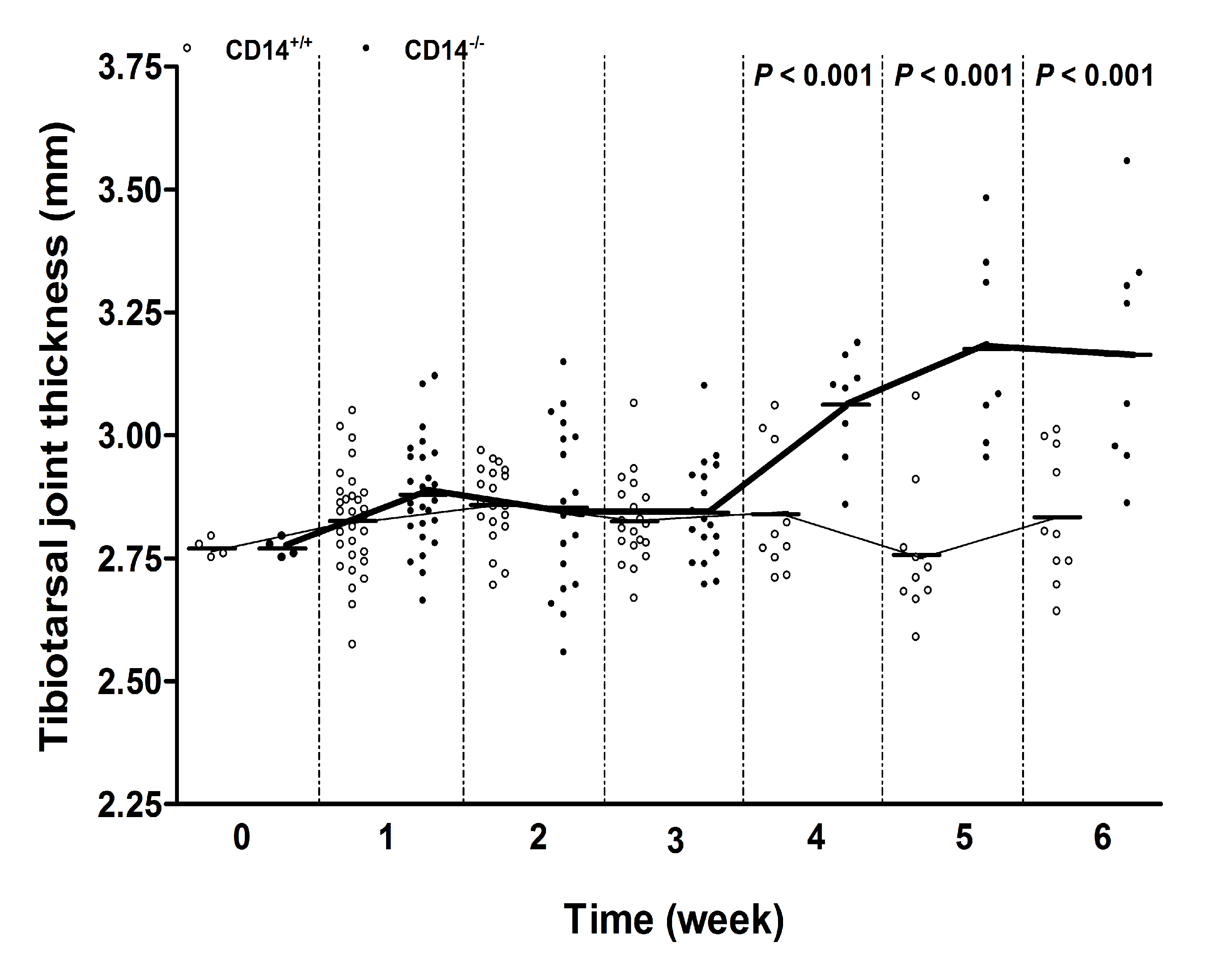

Supplement: Figure S1 — CD14 deficiency undermines the Lyme arthritis-resistant phenotype of C57BL/6 mice. CD14+/+ and CD14−/− C57BL/6 mice were infected using Ixodes scapularis ticks carrying B. burgdorferi and tibiotarsal joint thickness was measured at 1-wk intervals using digital calipers. The horizontal bars indicate mean thickness for each group and the data are representative of two independent experiments (n = 24). (0.56 MB TIF) [file ppat.1000687.s001.tif]

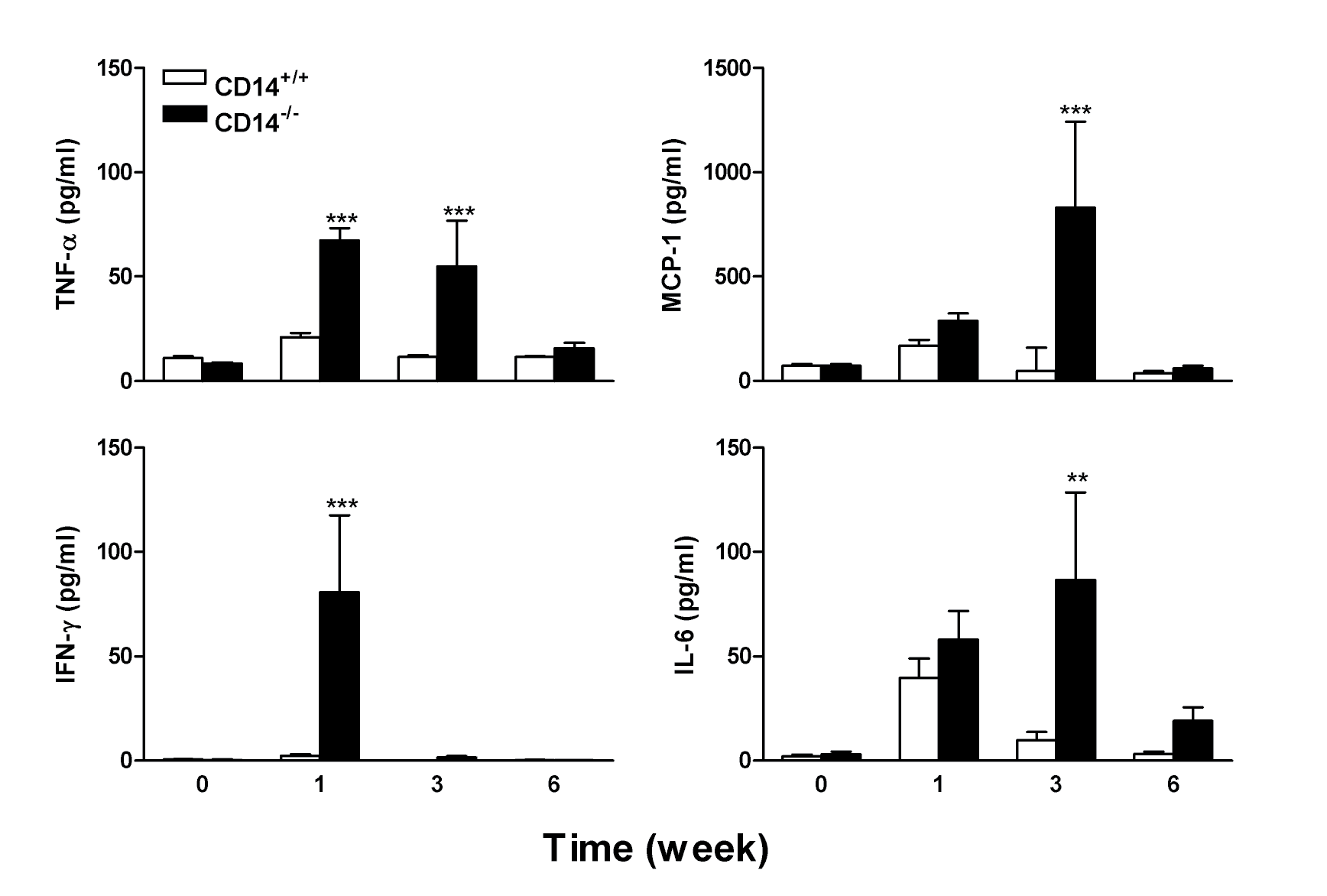

Supplement: Figure S2 — CD14 deficiency results in increased in vivo cytokine production in response to B. burgdorferi. CD14+/+ and CD14−/− C57BL/6 mice were tick-inoculated with B. burgdorferi and serum cytokine levels were measured using CBA at 1, 3, and 6 wks p.i.. Results represent mean±SEM from three independent experiments. **P<0.01, ***P<0.001. (0.22 MB TIF) [file ppat.1000687.s002.tif]
