# Supplementary material for: CD14 Signaling Restrains Chronic Inflammation through Induction of p38-MAPK/SOCS-Dependent Tolerance
Source: PLoS Pathog. 2009 Dec 11;5(12):e1000687. doi: 10.1371/journal.ppat.1000687 (PMC2781632; doi:10.1371/journal.ppat.1000687)
Supplement: Table S1 — CD14 deficiency impairs clearance of B. burgdorferi in C57BL/6 mice. Mice were sacrificed at 1, 3, and 6 wks post tick-inoculation and DNA was isolated from the indicated tissues for qPCR analysis as described in Figure 2C and Methods. Results represent mean±SEM from three independent experiments (n = 10 to 16) wherein samples were run in triplicate. *P<0.05, **P<0.01. (0.10 MB PDF) [file ppat.1000687.s003.pdf]

Table S1: CD14 deficiency impairs clearance of *B. burgdorferi* in C57BL/6 mice.

		Bladder	Heart	Ear	Joint
Day 7	$CD14^{+/+}$	2,146 ± 1,527	412 ± 187	24,887 ± 6,037	451 ± 304
	$CD14^{-/-}$	1,048 ± 584	466 ± 168	57,900 ± 15,587	113 ± 27
Day 21	$CD14^{+/+}$	359 ± 88	420 ± 178	1,923 ± 807	3,831 ± 1,304
	$CD14^{-/-}$	1,680 ± 748*	2,493 ± 1,014*	10,943 ± 6,760	8,871 ± 1,976*
Day 42	$CD14^{+/+}$	159 ± 34	52 ± 9	153 ± 36	692 ± 227
	$CD14^{-/-}$	407 ± 67**	164 ± 47**	1,016 ± 306**	1,780 ± 551

Mice (n = 10 to 16) were sacrificed at one, three and six weeks post tick-inoculation and DNA was isolated from the indicated tissues for qPCR analysis. Results represent the mean ± SEM of *B. burgdorferi flaB* copy numbers for each group of mice (normalized to $1x10^4$ copies of murine *nidogen*) from three independent experiments. Where indicated, differences between the $CD14^{+/+}$ and $CD14^{-/-}$ groups were considered significant using the nonparametric Mann Whitney test (*$P < 0.05$, **$P < 0.01$).
