# Supplementary material for: CD14 Signaling Restrains Chronic Inflammation through Induction of p38-MAPK/SOCS-Dependent Tolerance
Source: PLoS Pathog. 2009 Dec 11;5(12):e1000687. doi: 10.1371/journal.ppat.1000687 (PMC2781632; doi:10.1371/journal.ppat.1000687)
Supplement: Table S2 — Primer sequences used in qPCR. (0.10 MB PDF) [file ppat.1000687.s004.pdf]

Table S2. Primer sequences used in qPCR

Gene	5'/3'	Primer sequence
tlr2	5'	TTTCCACGGGCTGTGGTAC
	3'	TGGGCTTCCTCTTGGCC
tlr1	5'	TTCCGTGATGCACAGCTCC
	3'	CTCTGCTCGCCTGAGTTCTTC
tlr6	5'	ACCACAAGCTGCGGGT
	3'	CGTTTGCCCTTCTCAGTAGGC
socs1	5'	AGGATGGTAGCACGCAAC
	3'	GAAGACGAGGACGAGGAG
socs3	5'	AGGAGAGCGGATTCTACTG
	3'	TCACACTGGATGCGTAGG
cis	5'	AGGAAGTGACAGAGGAGAC
	3'	CCCGAAGGTAGGAGAACG
irakm	5'	ACGACCCTGGACCTCTGG
	3'	GGAAGTCTTTGTGGAAATGTTTGG
mkp1	5'	CCACTCAAGTCTTCTTTCTC
	3'	AAGGCGTCAAGCATATCC
irf1	5'	GTCGTCAGCAGCAGTCTC
	3'	TTCCTCATCCTCGTCTGTTG
irf3	5'	ATAAAGTGTATGAGTTTGTGAC
	3'	TGGCAGTTGTTGAGAAGG
irf7	5'	TTGGAGACTGGCTATTGG
	3'	ATCCCTACGACCGAAATG
stat1	5'	CACGCTGCCTATGATGTC
	3'	CCTGGAGATTACGCTTGC
stat3	5'	GGCACCTTGGATTGAGAG
	3'	TGCTGATAGAGGACATTGG
stat4	5'	CACTACCTGGACGGAGAG
	3'	CCATAGGAAAGTTGTCATCATAG
inos	5'	GTTAGAGACACTTCTGAGG
	3'	TTTGGTAGGATTTGACTTTG
18s RNA	5'	ATAGCTGTATATTAAAGTTG
	3'	GTCCTATTCCATTATTCC
